# A Novel Green Preparation of Ag/RGO Nanocomposites with Highly Effective Anticancer Performance

**DOI:** 10.3390/polym13193350

**Published:** 2021-09-30

**Authors:** Maqusood Ahamed, Mohd Javed Akhtar, M. A. Majeed Khan, Hisham A. Alhadlaq

**Affiliations:** 1 King Abdullah Institute for Nanotechnology, King Saud University, Riyadh 11451, Saudi Arabia; mjakhtar@ksu.edu.sa (M.J.A.); mmkhan@ksu.edu.sa (M.A.M.K.); hhadlaq@ksu.edu.sa (H.A.A.); 2 Department of Physics and Astronomy, College of Science, King Saud University, Riyadh 11451, Saudi Arabia

**Keywords:** Ag/RGO nanocomposites, green preparation, anticancer performance, potential mechanism, oxidative stress

## Abstract

The efficacy of current cancer therapies is limited due to several factors, including drug resistance and non-specific toxic effects. Due to their tuneable properties, silver nanoparticles (Ag NPs) and graphene derivative-based nanomaterials are now providing new hope to treat cancer with minimum side effects. Here, we report a simple, inexpensive, and eco-friendly protocol for the preparation of silver-reduced graphene oxide nanocomposites (Ag/RGO NCs) using orange peel extract. This work was planned to curtail the use of toxic chemicals, and improve the anticancer performance and cytocompatibility of Ag/RGO NCs. Aqueous extract of orange peels is abundant in phytochemicals that act as reducing and stabilizing agents for the green synthesis of Ag NPs and Ag/RGO NCs from silver nitrate and graphene oxide (GO). Moreover, the flavonoid present in orange peel is a potent anticancer agent. Green-prepared Ag NPs and Ag/RGO NCs were characterized by UV-visible spectrophotometry, transmission electron microscopy (TEM), scanning electron microscopy (SEM), energy dispersive spectroscopy (EDS), X-ray diffraction (XRD), and dynamic light scattering (DLS). The results of the anticancer study demonstrated that the killing potential of Ag/RGO NCs against human breast cancer (MCF7) and lung cancer (A549) cells was two-fold that of pure Ag NPs. Moreover, the cytocompatibility of Ag/RGO NCs in human normal breast epithelial (MCF10A) cells and normal lung fibroblasts (IMR90) was higher than that of pure Ag NPs. This mechanistic study indicated that Ag/RGO NCs induce toxicity in cancer cells through pro-oxidant reactive oxygen species generation and antioxidant glutathione depletion and provided a novel green synthesis of Ag/RGO NCs with highly effective anticancer performance and better cytocompatibility.

## 1. Introduction

Silver nanoparticles (Ag NPs), as one of the noble metals, possess unique physicochemical properties, including high thermal and electrical conductivity, high catalytic activity, good chemical stability, and surface-enhanced plasmon resonance effects [1,2]. Ag NPs also display excellent biological activities, e.g., broad-spectrum antimicrobial, antiviral, anti-inflammatory, and anticancer activities [3,4,5]. Additionally, due to their great optical properties, Ag NPs have also been used in electronics, catalysis, and biosensors [6]. However, the toxic potential of Ag NPs in human and environmental health are major hurdles to their biomedical and industrial applications [7,8]. The toxicity of Ag NPs has been reported in several in vitro and in vivo (mammalian and non-mammalian animals) studies [9,10,11].

Graphene derivatives, such as graphene oxide (GO) and reduced graphene oxide (RGO), have received great attention in the fields of electronics, sensing, and biomedicine due to their incredible physical and chemical features. RGO and its nanocomplex have been studied for antimicrobial, wound healing, drug delivery, and anticancer applications [12,13]. RGO surfaces have a large number of oxygen functional groups and surface defects, which makes them favourable for the development of nanocomposites (NCs) of RGO and metal/metal oxide for biomedical applications [14]. Currently, investigators are devoting a large amount of attention to the development of RGO and metal/metal oxide-based NCs due to their inherently superior biological activities that cannot be achieved by single composition [15,16,17].

Currently, NPs/NCs are being synthesized through three main routes: physical, chemical, and green methods [18,19]. Researchers are now recommending that the green method of NPs/NC synthesis is the best method due to its facile processing, use of non-toxic chemicals, and low cost [20,21,22]. The reducing and capping agents play important roles in the preparation of NPs/NCs. Highly toxic chemicals/solvents used in physical and chemical methods of NPs/NCs synthesis are responsible for environmental hazards [23,24]. Additionally, the use of toxic chemicals and solvents limits the application of NPs/NCs in medical and clinical fields [22]. Green synthesis requires the use of extracts from fruits, vegetables, or plants as reducing and stabilizing agents [25]. Biologically developed capping and reducing agents for the green synthesis of NPs/NCs are not harmful to the environment. Hence, the green method eliminates the use of expensive chemicals, consumes less energy, and produces eco-friendly NPs/NCs and by-products. However, it is still challenging to develop a simple, rapid, and inexpensive green protocol for the synthesis of Ag/RGO NCs with highly effective anticancer performance. 

The green synthesis of Ag/RGO NCs is gaining momentum [18,26,27]. It is advisable to prepare Ag/RGO NCs with highly effective anticancer performance and negligible side effects to humans and the environment. This study aimed to develop a simple, inexpensive, and environmentally friendly approach for the preparation of Ag/RGO NCs using orange (*Citrus sinensis*) peel extract. Oranges are among the most productive fruits worldwide, and orange peels, their main agricultural waste product, contain a large number of phytochemicals [28]. Orange peels contain polyphenols and polysaccharides that act as reducing agents, and carboxylic groups, amino acid and citric acid, which act as stabilizing agents [29]. The major active biological constituents in citrus fruits and peels are flavonoids [30]. The high concentrations of flavonoids present in orange peel extract have shown anticancer activity, as well as the prevention of infectious and degenerative diseases [31]. 

Orange peel extract was prepared by the maceration process [22,32]. A number of studies reported that the maceration process for the preparation of orange peel extract is an excellent method for the green synthesis of NPs [28,33,34]. Green-synthesized Ag NPs and Ag/RGO NCs were characterized by modern analytical techniques, such as transmission electron microscopy (TEM), scanning electron microscopy (SEM), energy dispersive X-ray spectroscopy (EDS), X-ray diffraction (XRD), and dynamic light scattering (DLS). The anticancer efficiency of Ag NPs and Ag/RGO NCs was examined in human breast cancer (MCF7) and human lung cancer (A549) cells. The cytocompatibility of prepared samples was assessed in human normal breast epithelial (MCF10A) cells and human normal lung fibroblasts (IMR90). Furthermore, the potential mechanisms of the anticancer activity of Ag/RGO NCs were delineated through the oxidative stress pathway. 

## 2. Materials and Methods

### 2.1. Preparation of Orange Peels Extract

Orange peels were obtained from locally purchased fresh orange fruit. Peels were washed with deionized water and dried in a food drier (12–15 h). Dried peels were ground into a fine powder by a locally purchased grinder. Then, 5 g of orange peel powder was soaked in 250 mL deionized water and continuously stirred for 5 h. Afterwards, the mixture was placed in a water bath (Cole-Parmer, Vernon Hills, IL, USA) at 60 °C for 2 h. At last, the mixture was filtered with filter paper (pore size 0.2 µm), and the resulting extract was stored at 4 °C for further application.

### 2.2. Synthesis of Ag NPs and Ag/RGO NCs

**Figure 1:**
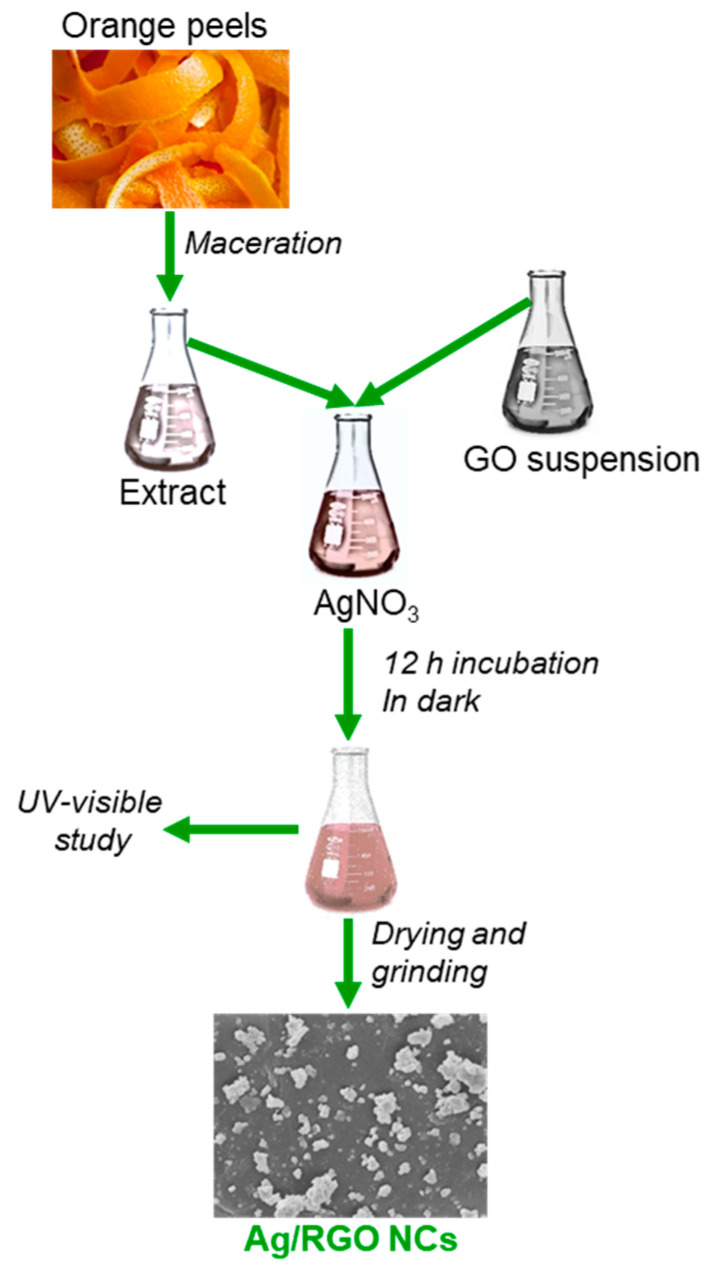
A schematic of green synthesis of Ag/RGO NCs using orange peel extract.

Silver nitrate (AgNO3, Millipore-Sigma, St. Louis, MO, USA), graphene oxide (GO, Millipore-Sigma), and orange peel extract were utilized as precursors for the synthesis of Ag NPs and Ag/RGO NCs. Briefly, an aqueous solution of 1 mM silver nitrate (1 mM) was prepared. Then, 50 mg of GO was also suspended in 100 mL of deionized water and kept in a water bath sonicator. The reaction was started by adding 20 mL of orange peel extract and 20 mL of GO suspension into 160 mL of aqueous solution of silver nitrate (1 mM) under mild stirring. The reaction mixture was incubated for 12 h in a dark setting at room temperature to avoid photo-activation of silver nitrate. After the completion of the incubation period, samples were dried at 90 °C for 3 h, and then ground into a fine powder for characterization and application. Pure Ag NPs were also prepared using the same method, without the addition of GO suspension. A schematic diagram of Ag/RGO NCs synthesis is presented in Figure 1.

### 2.3. Characterization of Ag NPs and Ag/RGO NCs

UV-visible spectra of green-prepared Ag NPs and Ag/RGO NCs was evaluated between 250 and 750 nm using the Shimadzu UV-1800 spectrophotometer. X-ray diffraction (XRD) (Pan Analytic X’Pert Pro, Malvern Instruments, Malvern, WR14, 1XZ, UK) equipped with Cu-Kα radiation (λ = 0.15405 nm, at 45 kV and 40 mA) was used to assess the crystallinity and phase-purity of green-prepared Ag NPs and Ag/RGO NCs. Morphological analysis, elemental mapping, and other structural characterization were further carried out by field emission transmission electron microscopy (FETEM) (JEM-2100, JEOL, Inc., Tokyo, Japan) and field emission scanning electron microscopy (FESEM) (JSM-7600F, JEOL, Inc., Tokyo, Japan). The characterization of NPs/NCs in aqueous suspension (hydrodynamic size and zeta potential) was carried out by dynamic light scattering (DLS) (ZetaSizer, Nano-HT, Malvern Instruments).

### 2.4. Cell Culture

Human breast cancer cells (MCF7), human lung cancer cells (A549), human normal breast epithelial cells (MCF10A), and human lung fibroblasts (IMR90) were purchased from American Type Culture Collection (ATCC, Manassas, WV, USA). Cells were cultured in Dulbecco‘s Modified Eagle’s Medium (DMEM) with the supplementation of 10% fetal bovine serum (FBS) and antibiotics (100 U/mL of penicillin and 100 µg/mL of streptomycin). Cells were grown at 37 °C in a humidified CO_2_ incubator (Heracell 150i, Thermo Fisher Scientific, Waltham, MA, USA) with 5% CO_2_ supply.

### 2.5. Exposure Procedure

The 1 mg/mL stock suspension of Ag NPs and Ag/RGO NCs was prepared in deionized water. Working concentrations (0.5–100 µg/mL) were diluted in culture medium. First, cells were exposed to different dosages (0.5–100 µg/mL) of Ag NPs and Ag/RGO NCs to examine their anticancer performance in a dose-dependent manner. Then, one moderate cytotoxic dosage (10 µg/mL) of each nanoscale material was chosen to explore potential mechanisms of anticancer activity through the oxidative stress pathway.

### 2.6. Anticancer Performance Assays

The anticancer activity of green-prepared NPs and NCs was examined by a tetrazolium dye 3-(4, 5-dimethylthiazol-2-yl)-2, 5-diphenyltetrazolium bromide (MTT) assay [35] with some specific modifications [36]. MTT assay is based on the principle that live cells are able to reduce yellow MTT salt into purple formazan crystals. These formazan crystals dissolved in acidified isopropanol, and absorbance was measured at 570 nm by a microplate reader (Synergy-HT, BioTek, Vinnoski, VT, USA). Potential mechanisms of anticancer activity of prepared samples were delineated by measuring the intracellular ROS and GSH levels. The ROS level was estimated using a cell-permeable probe 2′-7′-dichlorodihydrofluorescein diacetate (H_2_DCFDA) (Millipore-Sigma) [37]. Upon reaction with ROS, the non-fluorescent H_2_DCFDA was converted into highly fluorescent 2′-7′-dichlorofluorescein (DCF). The fluorescence intensity of DCF was measured at 485/520 nm (excitation/emission wavelength) using a microplate reader (Synergy-HT, BioTek). Ellman’s protocol was used to estimate the intracellular glutathione level (GSH) [38]. The intracellular level of GSH was represented as nmol GSH/mg protein. Protein assay was performed using Bradford’s method [39].

### 2.7. Statistical Analysis

One-way analysis of variance (ANOVA) followed by Dennett’s multiple comparison tests was applied to analyse the biochemical data. The *p* < 0.05 was assigned as statistically significant. All the biochemical data are represented as the mean ± SD of three independent experiments (*n* = 3).

## 3. Results and Discussion

### 3.1. UV-Visible Spectrophotometer Study

**Figure 2:**
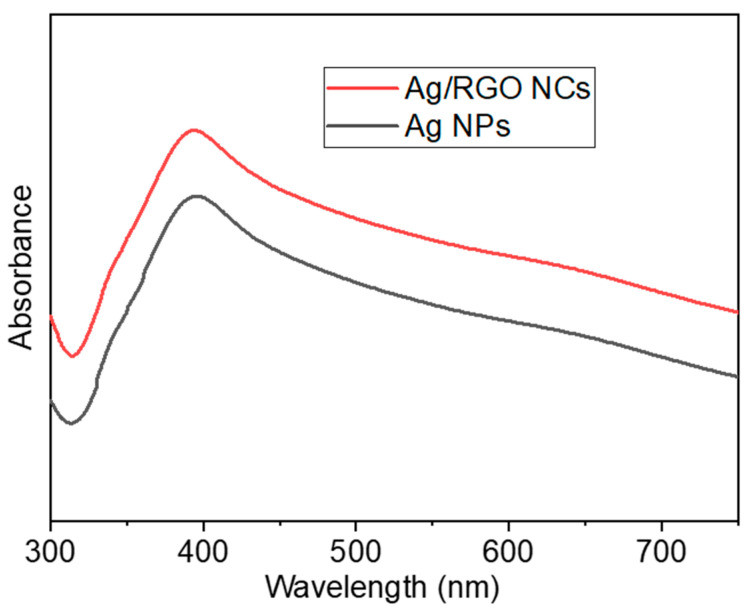
UV-visible spectra of Ag NPs and Ag/RGO NCs prepared from orange peel extract.

The colour of orange peel extract changed from light orange to dark brown after incubation with AgNO_3_ and GO for 12 h; the colour change reveals an indication of formation of Ag NPs and Ag/RGO NCs. The specific absorption peak of Ag NPs occurs in the range of 380–450 nm depending on shape, size, and agglomeration [40,41]. Hence, a UV-visible spectrophotometer was used to confirm the formation of Ag NPs and Ag/RGO NCs in the range of 250–750 nm. In the present study, Ag NPs and Ag/RGO NCs exhibited a strong plasma absorption band at ~395 nm (Figure 2). Our results were in agreement with those of other studies [42,43]. 

### 3.2. XRD Study

**Figure 3:**
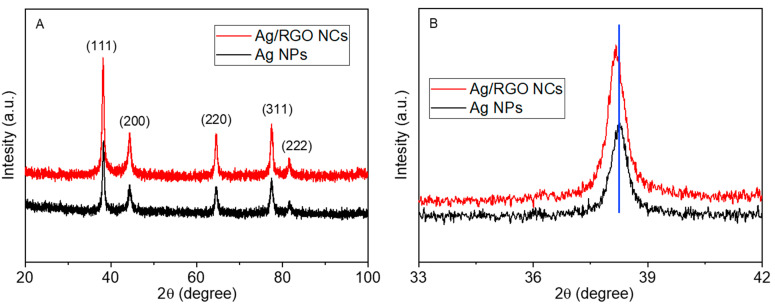
XRD characterization: (**A**) XRD spectra of Ag NPs and Ag/RGO NCs; (**B**) peak shifting.

XRD spectra of green-prepared Ag NPs and Ag/RGO NCs are given in Figure 3A. The five distinct diffraction peaks at 2θ = 38.16, 44.32, 64.52, 77.45, and 81.59 correspond to the crystal planes (111), (200), (220), (311), and (222), respectively, for the Ag/RGO NCs, which occurs with the face-centred cubic structure of metallic Ag (JCPDS card no.04-0783) [44]. The incorporation of RGO did not alter the original structure of metallic Ag as all the peaks of Ag/RGO NCs were similar to those of pure Ag NPs [45]. The absence of diffraction peaks of RGO in Ag/RGO NCs suggests that the uniform integration of Ag NPs inhibited the restacking of RGO sheets [46], and indicates the successful synthesis of Ag/RGO NCs. No other peaks attributed to impurity were identified, which indicates the high purity of the prepared samples.

Scherrer’s formula [47] was applied to calculate the particle size of the prepared nanoscale materials corresponding to prominent peak (111). The average particle sizes of pure Ag NPs and Ag/RGO NCs were around 13 and 9 nm, respectively. We further observed that Ag/RGO NCs showed a slight shift of the XRD peak (111) towards a lower value in comparison to pure Ag NPs (Figure 3B). The shifting of the peak toward a lower value further supports the successful formation of Ag/RGO NCs.

### 3.3. TEM Study

**Figure 4:**
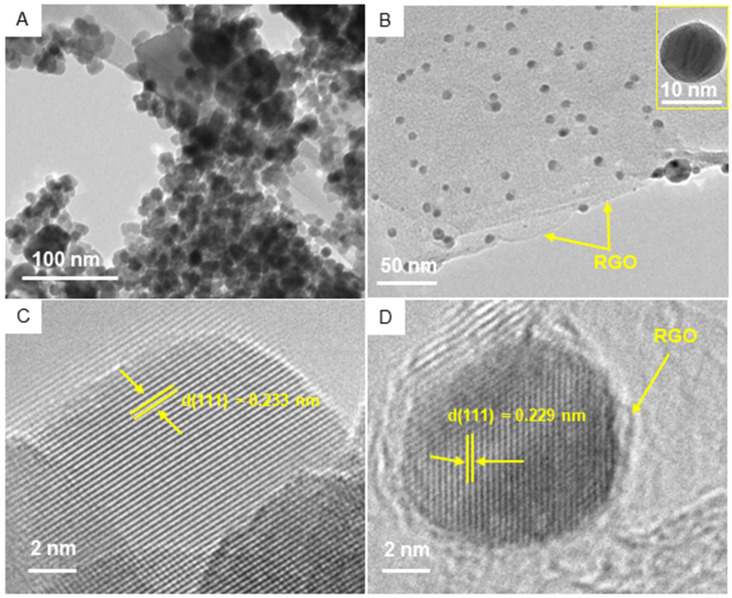
TEM characterization: (**A**) low-resolution TEM image of Ag NPs; (**B**) low-resolution TEM image of Ag/RGO NCs; (**C**) high-resolution TEM image of Ag NPs; (**D**) high-resolution TEM image of Ag/RGO NCs.

**Figure 5:**
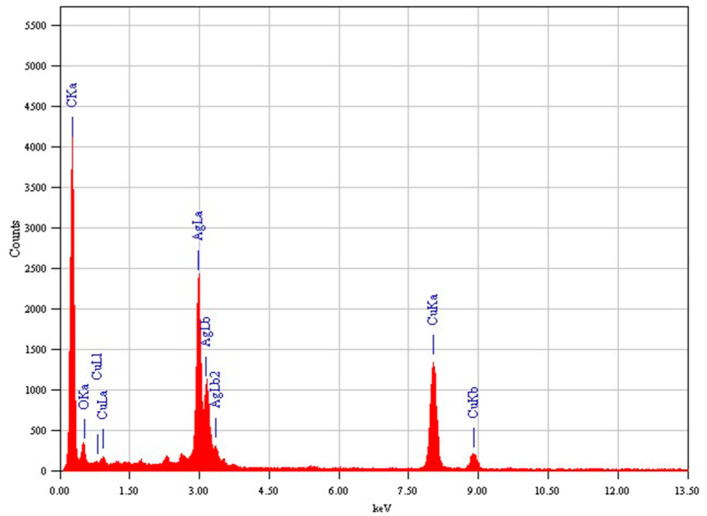
TEM-led EDS spectra of Ag/RGO NCs.

The TEM characterization of pure Ag NPs and Ag/RGO NCs is presented in Figure 4. Pure Ag NPs were nearly spherical with some degree of agglomeration (Figure 4A). In Ag/RGO NCs, Ag NPs were almost uniformly anchored on RGO sheets (Figure 4B). The particle sizes calculated from TEM were approximately 12 and 8 nm for pure Ag NPs and Ag/RGO NCs, respectively, which agreed with the sizes calculated from XRD. Ag NPs on RGO sheets were less agglomerated than pure Ag NPs. Moreover, Ag NPs acted as spacers to avoid the restacking of RGO sheets, and enhanced the surface area of NCs. This could be a possible reason for the particle size reduction of Ag/RGO NCs. Decrements in the particle size of NPs after the incorporation of RGO were also reported in other studies [16,46,48]. Nanoscale materials with a smaller size and higher surface area exhibited higher biological activity [26]. High-resolution TEM images (Figure 4C,D) show the clear lattice fringes with measured interplanar distances of 0.233 and 0.229 nm for pure Ag NPs and Ag/RGO NCs, respectively, which corresponds to the (111) plane of the face-centred cubic structure of Ag [26]. Elemental analysis of Ag/RGO NCs by TEM-led EDS indicated the presence of Ag, C, and O elements with no impurities (Figure 5). The presence of Cu peaks was due to the utilization of a Cu-based grid. 

### 3.4. SEM Study

**Figure 6:**
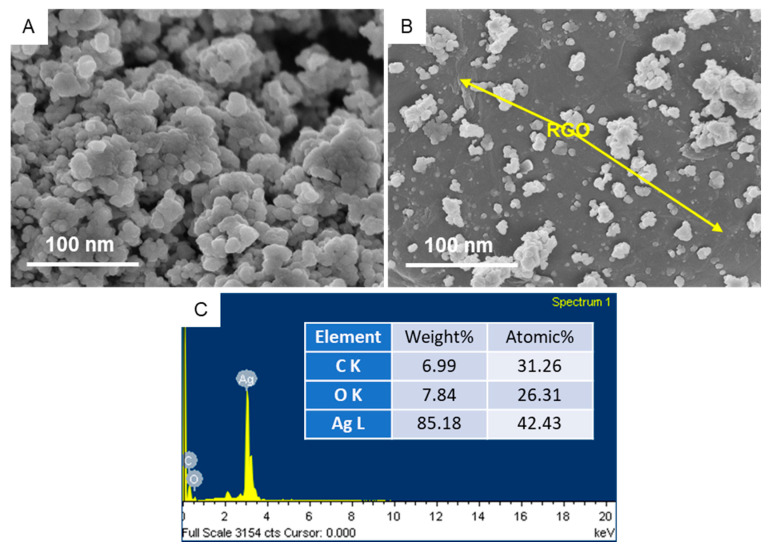
SEM characterization: (**A**) SEM image of Ag NPs; (**B**) SEM image of Ag/RGO NCs; (**C**) SEM-led EDS spectra of Ag/RGO NCs.

**Figure 7:**
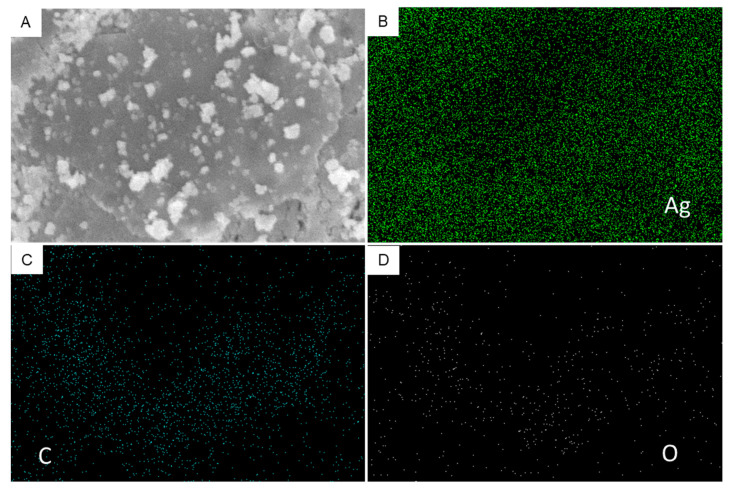
SEM elemental mapping of Ag/RGO NCs: (**A**) SEM micrograph; (**B**) Ag mapping; (**C**) C mapping; (**D**) O mapping.

Figure 6 shows the surface morphology and elemental composition of green-prepared samples. SEM images suggested that the smooth morphology of Ag NPs (Figure 6A) and Ag NPs were well embedded on the surface of RGO sheets (Figure 6B), which is supported by TEM micrographs. The implanted Ag NPs on RGO sheets created a strong interaction between them, resulting in the effective migration of charge carriers (electrons and holes) from the inner part of NCs to the surface. Hence, charge carriers can participate in surface redox reactions [49]. This phenomenon could be helpful in photocatalysis and cancer therapy [50]. The quantitative elemental composition of Ag/RGO NCs is presented in Figure 6C. The presence of Ag, C, and O elements in Ag/RGO NCs was in agreement with TEM-led EDS data. Figure 7 shows the elemental mapping of Ag/RGO NCs, which further confirmed the homogenous distribution of Ag, C, and O in Ag/RGO NCs.

### 3.5. DLS Study

It is essential to examine the aqueous behaviour of nanomaterials (e.g., surface charge, particle distribution, and stability) before their biological activity assessments [51,52]. DLS is an important tool to assess the aqueous behaviour of nanoscale materials [53]. In this study, DLS data demonstrated that the hydrodynamic sizes of pure Ag NPs and Ag/RGO NCs in deionized water and culture medium were several times higher (43–65 nm) than particle sizes estimated from XRD and TEM (Table 1). This may be ascribed to the fact that DLS measures the Brownian motion, and the subsequent size distribution of a group of NPs/NCs in aqueous suspension provides an average hydrodynamic size. During the DLS study, there was a tendency of NPs/NCs to agglomerate in aqueous suspension, thereby showing the size of clumped NPs/NCs rather than individual NPs/NCs [53,54].

Zeta potential data suggested that colloidal suspensions of Ag NPs and Ag/RGO NCs in deionized water and culture medium were fairly stable, as these values ranged from 21 to 28 mV (Table 1). A higher value of zeta potential (either positive or negative) is directly proportional to the greater stability of colloidal suspension [55]. Additionally, positive surface charges (zeta potential value) of Ag NPs and Ag/RGO NCs offer encouraging conditions for their interaction with negatively charged cancer cells [56].

### 3.6. Anticancer Study

**Figure 8:**
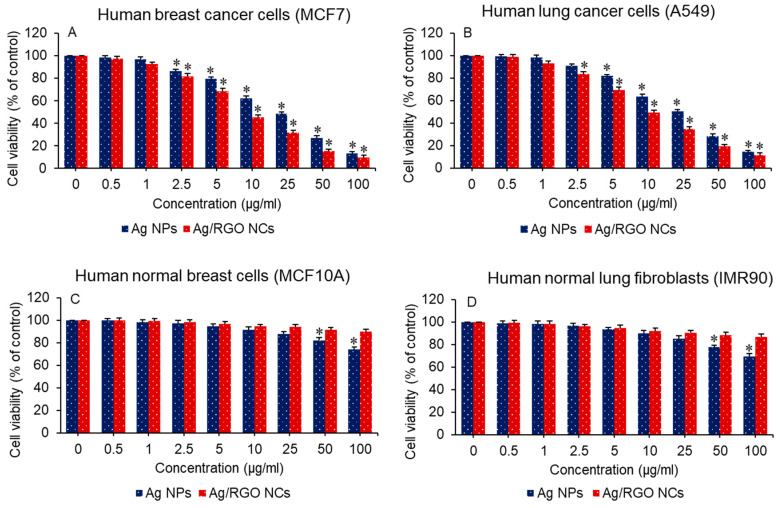
Anticancer performance of Ag NPs and Ag/RGO NCs in cancer cells. * *p* < 0.05 statistically different from control (0 concentration of NPs/NCs).

The anticancer performance of green-synthesized Ag NPs and Ag/RGO NCs was studied in two different types of cancer cells: human breast cancer (MCF7) and human lung cancer (A549) cells. Both types of cancer cells were treated with different concentrations of Ag NPs and Ag/RGO NCs, and anticancer performance was evaluated by MTT assay. Results showed that pure Ag NPs and Ag/RGO NCs kill both types of cancer cells in a dose-dependent manner (Figure 8A,B). Furthermore, the killing potential of Ag/RGO NCs against both cancer cells was twice that of pure Ag. The IC_50_ values of Ag/RGO NCs (10 µg/mL for MCF7 and 11 µg/mL for A549) were almost half of those of pure Ag NPs (19 µg/mL for MCF7 and 20 µg/mL for A549) (Table 2). The high anticancer efficacy of Ag/RGO NCs might be due to excellent green mediated (orange peel components) synergism between the two functional materials, Ag and RGO. Earlier reports suggested that bioactive flavonoid present in orange peel extract is a potent anticancer agent [31]. Therefore, orange peel extract-mediated green-synthesized Ag/RGO NCs have the potential to act as a chemotherapeutic drug. The high anticancer efficacy of Ag and graphene derivative-based NCs synthesized by different methods has also been reported by other studies. For example, Gurunathan and co-workers observed that chemically prepared RGO-Ag NCs showed higher cytotoxicity in ovarian cancer (A2780) than pure GO, RGO, and Ag NPs [27]. Another study also demonstrated that green-prepared (walnut husk) Ag-GO NCs exerted higher cytotoxicity to MCF7 cells in comparison to pure Ag NPs [57].

The application of anticancer drugs depends on their biocompatibility with normal cells/tissues. In this study, the cytotoxicity of Ag NPs and Ag/RGO NCs was examined in the normal counterparts of the above cancer cells: human normal breast epithelial (MCF10A) cells and human normal fibroblasts (IMR90). Results showed that green-synthesized pure Ag NPs and Ag/RGO NCs did not induce cytotoxicity to both types of normal cells (MCF10A and IMR90) (Figure 8C,D). Moreover, the cytocompatibility of Ag/RGO NCs in both normal cells was higher in comparison to pure Ag NPs. Overall, the anticancer study indicated that green-synthesized Ag/RGO NCs exhibited a higher potential of anticancer activity and better cytocompatibility than those of pure Ag NPs. Bioactive compounds present on green-prepared Ag NPs and Ag/RGO NCs might prevent their toxicity to normal cells. 

### 3.7. Potential Mechanisms of Anticancer Activity

**Figure 9:**
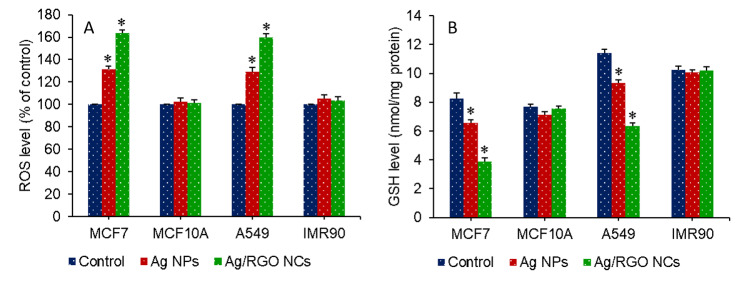
Oxidative stress response of cancer and normal cells against 10 µg/mL of Ag NPs and Ag/RGO NCs for 24 h: (**A**) ROS generation; (**B**) GSH depletion. * *p* < 0.05 statistically different from control.

Oxidative stress has been suggested as a potential mechanism of the anticancer response of green-prepared Ag NPs. [46]. In the present study, the anticancer mechanism of green-prepared present nanoscale materials was delineated through assessing the oxidative stress pathway. Pro-oxidant ROS and antioxidant GSH were assessed in cancer and normal cells after exposure for 24 h to 10 µg/mL of pure Ag NPs and Ag/RGO NCs. Figure 9 demonstrates that pure Ag NPs and Ag/RGO NCs induced intracellular ROS generation and GSH depletion in both types of cancer cells (MCF7 and A549). However, Ag NPs and Ag/RGO NCs were not able to affect ROS and GSH levels in either of the normal cells (MCF10A and IMR90). Additionally, the oxidative stress-generating potential of Ag/RGO NCs was greater than pure Ag NPs, which supports the cytotoxicity data.

**Figure 10:**
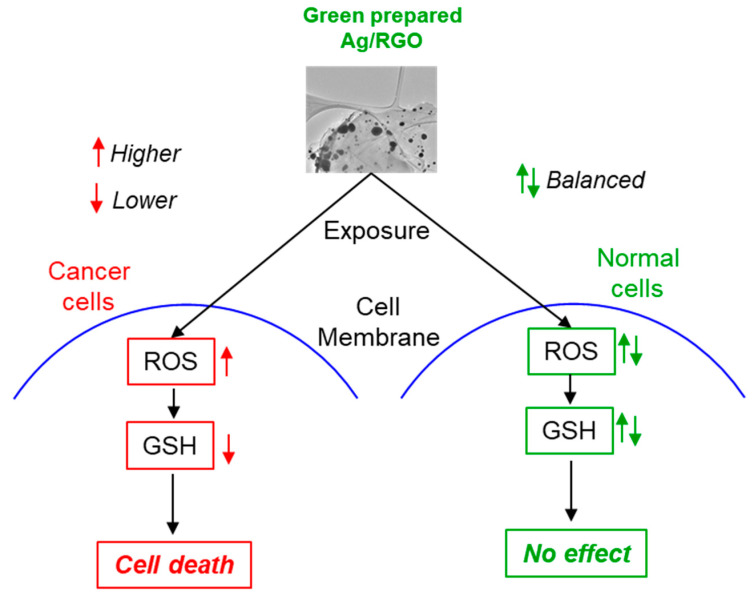
Possible mechanism of anticancer performance of Ag/RGO NCs.

The integration of RGO makes two crucial modifications in the physicochemical properties of Ag NPs, which play important roles in improving the anticancer performance of Ag/RGO NCs: (i) The firmly anchored Ag NPs on RGO sheets create a strong interaction between them that leads to an easy electron transfer process on the surface of NCs, resulting in highly effective anticancer performance through the intracellular generation of ROS [49]. (ii) The homogeneous anchoring of AG NPs on RGO sheets decreases the particle size and increases the surface of NCs. Smaller NPs generate greater intracellular ROS in comparison to higher NPs [58]. The oxidative stress-mediated anticancer activity of other nanoscale materials has also been proposed [50,59]. For example, our recent studies indicated that ZnO/RGO NCs and Zn-doped Bi_2_O_3_ NPs displayed anticancer activity through ROS generation [32,60,61]. The possible mechanism of anticancer performance in Ag/RGO NCs is depicted in Figure 10. 

## 4. Conclusions

A simple, cost-effective, and eco-friendly procedure was developed to prepare Ag NPs and Ag/RGO NCs. Green-synthesized Ag NPs and Ag/RGO NCs were characterized by UV-vis, XRD, TEM, SEM, EDS, and DLS techniques. XRD data confirm that the synthesis of face-centred cubic structures of metallic Ag and RGO implantation did not change the original crystal structure of Ag. A high-resolution TEM micrograph of Ag/RGO NCs indicated the presence of Ag and RGO with fine-quality lattice fringes without distortion. EDS elemental composition and mapping depicted the uniform presence of Ag, O, and C in Ag/RGO NCs. The DLS study demonstrated the outstanding colloidal stability of Ag NPs and Ag/RGO NCs. The anticancer study showed that the killing potential of Ag/RGO NCs against cancer (MCF7 and A549) was two-fold that of pure Ag NPs. Additionally, the cytocompatibility of Ag/RGO NCs in normal counterparts (MCF10A and IMR90) was higher than that of Ag NPs. Mechanistic data indicated that the anticancer activity of Ag NPs and Ag/RGO NCs was mediated through ROS generation and GSH depletion. Current work suggests a novel approach for highly effective cancer treatment through green-prepared Ag/RGO NCs. Further research on the antitumor efficacy of Ag/RGO NCs in animal models is warranted.

## Figures and Tables

**Table 1:** Dynamic light scattering (DLS) characterization of Ag NPs and Ag/RGO NCs.

NPs/NCs	Hydrodynamic Size (nm)		Zeta Potential (mV)	
	Deionized water	Culture medium	Deionized water	Culture medium
Ag NPs	59.8 ± 2.8	65.2 ± 3.3	25.7 ± 1.5	21.3 ± 1.3
Ag/RGO NCs	43.4 ± 1.6	46.7 ± 2.3	28.3 ± 0.9	23.7 ± 1.1

**Table 2:** IC_50_ values of pure Ag NPs and Ag/RGO NCs for human cancer cells.

NPs/NCs	Human Breast Cancer MCF7 Cells	Human Lung Cancer A549 Cells
Ag NPs	18.8 µg/mL	20.3 µg/mL
Ag/RGO NCs	9.7 µg/mL	10.9 µg/mL
